# Percutaneous tracheostomy in Costa Rican intensive care units: multicenter epidemiology, practices, and immediate complications

**DOI:** 10.62838/jccm-2026-0021

**Published:** 2026-07-27

**Authors:** Pablo Alvarez-Aguilar, Leonardo Chacón-Prado, Dennis Rojas-Quirós, Oscar Palma-Rodríguez, Donato Salas-Segura, TRAQUEOSCR Group

**Affiliations:** Hospital México, Caja Costarricense de Seguro Social, San José, Costa Rica; Hospital Rafaél Ángel Calderón Guardia, Caja Costarricense de Seguro Social, San José, Costa Rica

**Keywords:** airway management, tracheostomy, bedside tracheostomy, percutaneous tracheostomy, critical care, patient safety

## Abstract

**Introduction:**

This study describes the epidemiological and clinical profile of ICU patients undergoing percutaneous tracheostomy in Costa Rica and identifies predictors of acute complications, addressing ongoing debates on timing, technique, and risk stratification.

**Methods:**

We performed a prospective multicenter cohort study in eight CCSS hospitals (2019–2022), including adult ICU patients undergoing percutaneous tracheostomy. Demographic, clinical, and procedural data were collected, and multivariable logistic regression identified predictors of complications.

**Results:**

A total of 516 patients were analyzed (mean age 53.2 ± 16.3 years; 68.2% male). The main indications were anticipated prolonged ventilation (32.4%), neurological deficits (26.7%), and ventilation >10 days (21.8%). The Ciaglia and Griggs techniques were used in 51.0% and 48.3% of cases, respectively. Capnography was applied in 74.2%, ultrasound in 17.7%, and bronchoscopy in 3.1%. First-pass success was achieved in 85.1%. Acute complications occurred in 28.3% of patients, predominantly minor bleeding (25.4%), while serious complications (airway loss, false passage, or bleeding requiring surgery) were rare (3.9%). No procedure-related deaths were observed. Independent predictors of complications included anticoagulation (OR 2.82), obesity (OR 2.10), coagulopathy (OR 2.29), prior neck surgery (OR 3.49), cervical immobilization (OR 4.68), and technical difficulty (OR 4.15 for any complication; OR 2.00 for serious complications). Airway management by physicians, compared with respiratory therapists, was also associated with higher risk (OR 1.52).

**Conclusions:**

Percutaneous tracheostomy was feasible in multiple ICUs of the CCSS with complication rates comparable to international cohorts. Risk factors for complications included anticoagulation and prior neck surgery. Wider adoption of adjunctive monitoring tools and structured multidisciplinary training may further enhance procedural safety. These findings should be interpreted in the context of an observational design and a broad definition of complications.

## Introduction

A high proportion of ICU patients require translaryngeal airway placement for mechanical ventilation, airway protection, or relief of obstruction [1]. Tracheostomy is among the most common ICU procedures and has been performed for millennia [2,3,4]. Despite its long history, the optimal timing, indications, and preferred technique remain debated [2].

Since Jackson’s description of surgical tracheostomy in 1909, percutaneous techniques such as Ciaglia’s dilatational method and the Griggs forceps approach have become widely adopted [3,5]. These methods demonstrate complication rates similar or lower than surgical tracheostomy, and are considered safe across diverse critical care populations [6,7,8]. Advances in instruments, standardization, and adjuncts such as ultrasound have further improved safety, while operator experience remains essential [2,9,10,11].

Complications may occur intra-procedurally, during cannula maintenance, or after decannulation. In Costa Rica, a single-center study of 70 cases showed the procedure to be safe when performed by intensivists with bronchoscopic guidance [12]. However, multicenter evidence in the region is lacking. Recent multicenter data emphasize the variability in indications and timing of percutaneous tracheostomy across ICUs, underlining the importance of local epidemiological studies [13].

The objective of this study was to describe the incidence, types, and risk factors for complications associated with percutaneous bedside tracheostomy in Costa Rican ICUs. We hypothesized that percutaneous tracheostomy performed by trained intensivists would demonstrate an acceptable safety profile comparable to that reported in international series. Unlike most published cohorts, this study reflects real-world practice in a middle-income healthcare system with heterogeneous access to procedural adjuncts.

## Materials and methods

This observational cohort (TraqueosCR) was designed and reported following the STROBE Statement.

### Study design and setting

We conducted a prospective, multicenter cohort study in the intensive care units (ICUs) of hospitals within the Costa Rican Social Security Fund (Caja Costarricense de Seguro Social, CCSS). The study commenced in February 2019 and spanned 4 years. Participating centers included Enrique Baltodano (Liberia), Max Peralta (Cartago), Monseñor Sanabria (Puntarenas), Rafael Ángel Calderón Guardia, San Juan de Dios, San Rafael (Alajuela), and Tony Facio (Limón). The study period overlapped with the COVID-19 pandemic, during which ICUs experienced variable strain on resources and staff reallocation. Despite these challenges, percutaneous tracheostomy remained part of routine care, with adaptations in PPE use and workflow but without major changes in procedural technique. Although data collection was prospective, the analytical approach was retrospective, as hypotheses and regression models were defined after completion of data collection

### Participants

Inclusion criteria were: (1) adults ≥18 years; (2) ICU admission at a CCSS hospital; (3) bedside percutaneous tracheostomy performed by ICU personnel as part of routine care. Exclusion criteria were: (1) tracheostomies performed in the operating room; (2) procedures conducted by non-ICU personnel; (3) incomplete clinical records preventing analysis. During the study period, surgical tracheostomy was also performed in patients with distorted anatomy, uncontrolled coagulopathy, or other contraindications to bedside percutaneous tracheostomy; these cases were not included in the present analysis.

### Variables and definitions

“Trained intensivists” were defined as board-certified critical care specialists or fellows under direct supervision, with a minimum of 10 supervised tracheostomies, as per CCSS credentialing requirements. Operator experience was collected as a variable.

Obesity was defined as BMI ≥30 kg/m^2^; short neck as a thyromental distance <5 cm; and goiter as a palpable/enlarged thyroid. Coagulopathy was defined as INR >1.5, platelet count <100,000/mm^3^, or aPTT >45 s. Cervical immobility included rigid cervical collars, halo fixation, or recent cervical spinal surgery. Emergency tracheostomy was defined as a procedure performed without standard preparation (fasting, coagulation checks, or full monitoring) due to acute airway compromise. Technical difficulty was recorded as any anatomical or procedural challenge (short neck, obesity, poor landmarks, >2 puncture attempts, or difficult dilatation). Serious technical difficulty refers to failed cannulation, airway loss, false passage, or device damage.

First-pass success was defined as successful tracheal cannulation at the first needle puncture without the need for repositioning or additional attempts. Capnography was defined as continuous waveform end-tidal CO_2_ monitoring during needle insertion and cannula placement.

Complications were defined broadly to include minor events managed conservatively at the bedside, which may inflate overall complication rates compared with series using more restrictive definitions.

When available, percutaneous tracheostomy kits were recorded by brand/model (e.g., Ciaglia Blue Rhino^®^ [Cook Medical] and Griggs guidewire dilating forceps [Portex^®^/Smiths Medical]). For ultrasound, centers used a high-frequency linear probe (typically 7–15 MHz) for pre-procedural neck scanning when available.

### Data sources and collection

ICU teams prospectively completed a standardized electronic case report form. Data were stored in password-protected cloud storage; only the principal investigator retained access to the re-identification key. Analytic datasets were de-identified.

### Bias control

We minimized selection bias by including all consecutive eligible patients. Information bias was reduced through standardized definitions, obligatory CRF fields, and verification of key procedural variables against ICU logbooks. Missing data were treated as non-random and not imputed; analyses used available cases after internal consistency checks.

### Sample size

No formal sample size or power calculation was performed. The cohort included all consecutive eligible procedures across participating ICUs, providing a descriptive epidemiological overview rather than a hypothesis-driven trial.

### Statistical analysis

Continuous variables were tested for normality using the Shapiro–Wilk test. Normally distributed data are presented as mean ± standard deviation (SD), and non-normal data as median with interquartile range (IQR). Categorical variables are expressed as n (%). Comparisons between groups were performed using Student’s t-test or Mann–Whitney U test for continuous variables, and χ^2^ or Fisher’s exact test for categorical variables, as appropriate. To identify independent predictors of overall and major complications, multivariable logistic regression models were fitted adjusting for prespecified covariates (age, sex, SOFA score, indication, technique/adjuncts, coagulation profile, operator experience). Odds ratios (OR) with 95% confidence intervals (CI) were estimated. A two-sided p < 0.05 was considered statistically significant. Covariates included in multivariable models were prespecified based on clinical relevance and prior literature, rather than selected through automated variable selection procedures

All analyses were performed with Stata/MP 18.0 (StataCorp, College Station, TX, USA).

### Ethical considerations

The study was approved by the Institutional Review Board of the CCSS (approval number R018-SABI-00205). Informed consent was waived because this was an observational study with prospectively collected routine-care data and de-identified analysis, with no impact on clinical management.

## Results

### Participants

During the observation period, data were collected from 516 patients across eight participating centers. Baseline characteristics are presented in Table 1. Extended baseline characteristics by hospital are available in Table S1 (Supplementary Material). All included patients underwent bedside percutaneous tracheostomy and were followed until ICU discharge or death.

**Table 1.** Epidemiological characteristics of patients undergoing percutaneous tracheostomy in intensive care units of the Costa Rican Social Security Fund (CCSS), 2019–2022.

**Characteristics**	
Age (years)	
Mean (SD)	53.15 (16.28)
Median (Interquartile Range 25–75)	55 (41.5–65)

Men (%) / Women (%)	352 (68.2) / 164 (31.8)

Weight (kg)	
Mean (SD)	78.67 (17.73)
Median (Interquartile Range 25–75)	78 (67–89)

Height (cm)	
Mean (SD)	166.5 (9.20)
Median (Interquartile Range 25–75)	168 (160–173)

SOFA score at admission (n (%))	N:443
0–6	141 (31.8)
7–9	132 (29.8)
10–12	123 (27.8)
13–14	30 (6.8)
Greater than 15	17 (3.8)

Reason for ICU admission (n (%))	
Pneumonia	57 (11)
Polytrauma	51 (9.9)
Traumatic brain injury	86 (16.7)
Intracranial hemorrhage	51 (9.9)
Postoperative neurosurgical care	41 (7.9)
Acute respiratory distress syndrome	20 (3.9)
Ischemic stroke	11 (2.1)
Intra-abdominal infection	14 (2.7)
Postoperative abdominal surgery	7 (1.4)
Postoperative cardiac surgery	4 (0.8)
COVID-19	47 (9.1)

### Descriptive data

At ICU admission, 269 patients (52%) met criteria for shock, 283 (54.8%) required vasopressors, 55 (10.6%) required inotropes, and 465 (90%) were already on invasive mechanical ventilation. The distribution of reasons for ventilatory support and indications for tracheostomy are summarized in Table S2 (Supplementary Material).

Ventilatory parameters at the time of tracheostomy, including duration of mechanical ventilation, endotracheal tube size, FiO_2_ requirements, and respiratory index, are summarized in Table 2.

**Table 2.** Ventilatory parameters of patients undergoing percutaneous tracheostomy in intensive care units of the Costa Rican Social Security Fund (CCSS), 2019–2022.

**Variable**	**Distribution**
Duration of mechanical ventilation at the time of tracheostomy (days)	
Mean (SD)	7.02 (4.89)
Median (Interquartile Range 25–75)	6 (4–9)

Endotracheal tube internal diameter (F), n (%)	
6.5	1 (0.19)
7	54 (10.47)
7.5	302 (58.53)
8	150 (29.07)
8.5	8 (1.55)
9	1 (0.19)

FiO_2_ on the day of tracheostomy (%)	
Mean (SD)	42.26 (18.46)
Median (Interquartile Range 25–75)	35 (30–50)

Respiratory index on the day of the procedure (n = 214)	
Mean (SD)	252.55 (96.95)
Median (Interquartile Range 25–75)	255 (182–312)

**Fig. 1.**
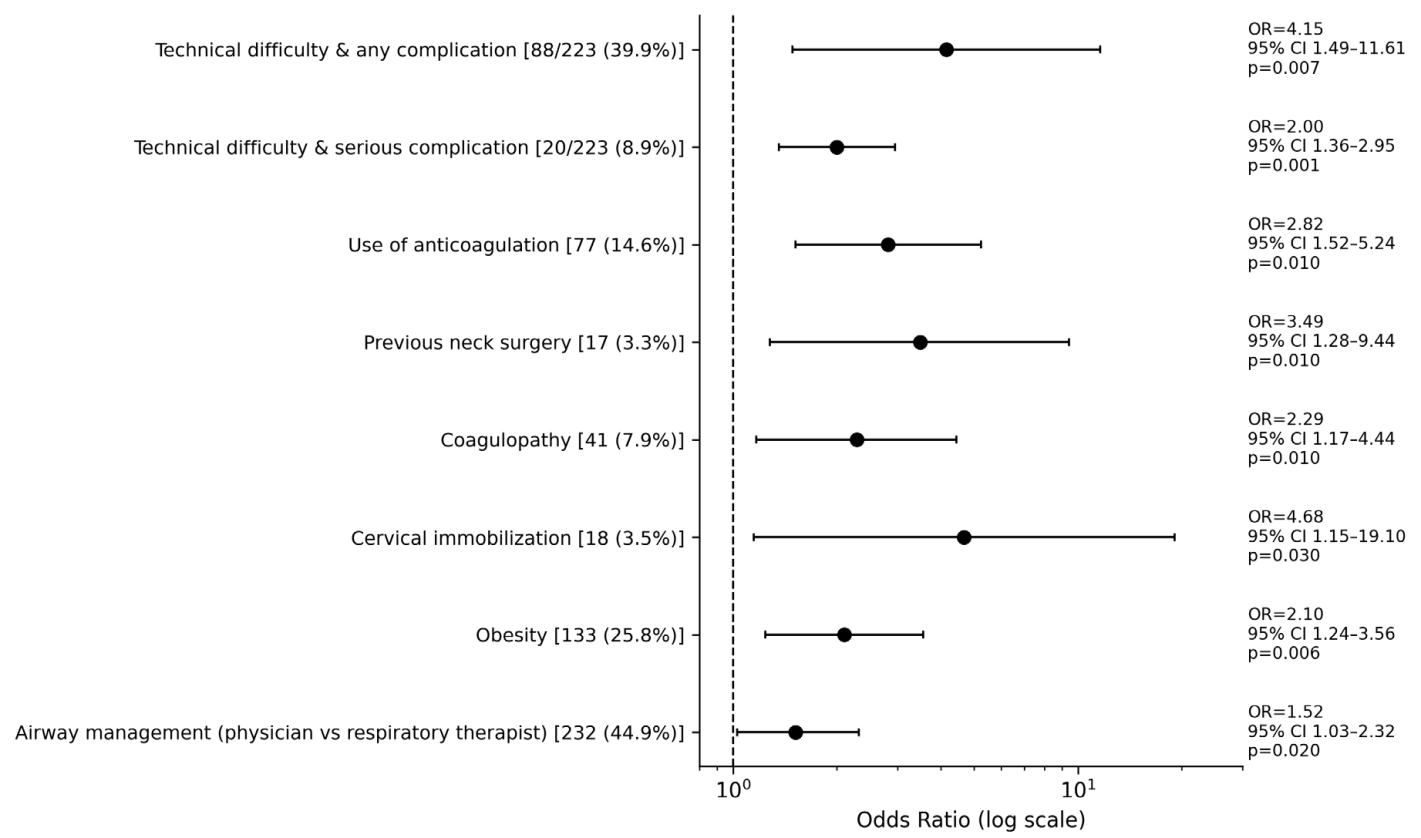
Forest plot of odds ratios (OR) and confidence intervals for immediate complications in percutaneous tracheostomy in intensive care units of the Costa Rican Social Security Fund (CCSS), 2019–2022.

During the procedure, FiO_2_ was increased to 100% in 473 cases (92.7%), and fasting was ordered in 340 cases (65.9%) with a mean duration of 4.26 ± 9 hours (median 2 h, IQR 0–6). Figure 1 shows the distribution of indications for tracheostomy.

At least one technical difficulty was reported in 223 patients (43.2%) (Table 3).

**Table 3.** Clinical parameters associated with greater difficulty in performing percutaneous tracheostomy in patients from intensive care units of the Costa Rican Social Security Fund (CCSS), 2019–2022.

**Characteristic**	**(%)**
Short neck	115 (22.3)
Coagulopathy	41 (7.9)
Previous neck surgery	17 (3.3)
Goiter	9 (1.7)
Neck burn	4 (0.8)
Cervical immobilization	18 (3.5)
Obesity	133 (25.8)
Procedure performed in an emergency situation	13 (2.5)

Most procedures were performed by critical care specialists (74.8%) or by residents under supervision (20.7%). A bronchoscope was used in 16 procedures (3.1%), with one incident of bronchoscope puncture. Ultrasound was used for neck scanning in 66 patients (17.7%), while intra-procedural ultrasound was applied in only 3 cases (0.9%).

Emergency procedures, though relatively rare (2.5%), were associated with omission of routine preparation steps such as coagulation checks and fasting.

While not statistically associated with increased complications, their inclusion highlights the need for clear contraindications, as most guidelines discourage percutaneous tracheostomy under emergency conditions.

Regarding puncture attempts, 439 procedures (85.1%) succeeded on the first attempt, 55 (10.7%) on the second, 18 (3.5%) on the third, 3 (0.6%) on the fourth, and 1 (0.2%) required a fifth attempt.

Pharmacologic strategies are described in Table S4 (Supplementary Material). Capnography was used in 383 patients (74.2%), and post-procedural chest radiography was requested in 96.7% (n = 499).

### Outcome data

Overall, 146 patients (28.3%) experienced at least one complication (Table 4). Bleeding-related events were the most common (131 patients, 25.4%), with 7 cases (1.4%) requiring surgical management. Potentially severe complications (excluding minor bleeding, bleeding controlled with sutures, isolated desaturation, and bronchoscope puncture) occurred in 20 patients (3.9%). Guidewire loss occurred in two procedures (0.39%). In both cases, the wire was accidentally dropped outside the sterile field and replaced without patient harm. No intratracheal wire migration was reported.

**Table 4.** Immediate complications associated with percutaneous tracheostomy in patients from intensive care units of the Costa Rican Social Security Fund (CCSS), 2019–2022.

**Complication**	**n (%)**
Bleeding controlled by pressure	93 (18.02)
Bleeding controlled with suture	31 (6.01)
Bleeding requiring surgical intervention	7 (1.36)
Airway loss	7 (1.36)
Desaturation	20 (3.88)
Pneumothorax	2 (0.39)
False passage	7 (1.36)
Pneumomediastinum	1 (0.19)
Subcutaneous emphysema	2 (0.39)
Posterior tracheal wall injury	1 (0.19)
Guidewire loss	2 (0.39)
Failed tracheostomy	2(0.39)

**Table 5.** Factors associated with immediate complications during percutaneous tracheostomy in patients from intensive care units of the Costa Rican Social Security Fund (CCSS), 2019–2022.

**Factors Associated with Complications**	**n (%)**	**OR**	**95% CI**	**p**
Technical difficulty and any complication	88/223 (39.91)	4.15	1.49–11.61	0.007
Technical difficulty and serious complication	20/223 (8.9)	2.00	1.36–2.95	0.001
Use of anticoagulation	77 (14.56)	2.82	1.52–5.24	0.01
Previous neck surgery	17 (3.3)	3.49	1.28–9.44	0.01
Coagulopathy	41 (7.9)	2.29	1.17–4.44	0.01
Cervical immobilization	18 (3.5)	4.68	1.15–19.1	0.03
Obesity	133 (25.8)	2.1	1.24–3.56	0.006
Airway management by medical staff vs. respiratory therapist	232 (44.9)	1.52	1.03–2.32	0.02

### Main results

In the multivariable logistic regression analysis, several factors were independently associated with the occurrence of complications (Factors associated with complications are summarized in Table 5 and illustrated in Figure 1). Patients receiving anticoagulation had a significantly higher risk (OR 2.82, 95% CI 1.52–5.24, p = 0.01), as did those whose airway was managed by medical staff rather than respiratory therapy personnel (OR 1.52, 95% CI 1.03–2.32, p = 0.02). The presence of any technical difficulty during the procedure was strongly associated with complications (OR 4.15, 95% CI 1.49–11.61, p = 0.007), while serious technical difficulty was associated with an increased risk of serious complications (OR 2.00, 95% CI 1.36–2.95, p = 0.001). Clinical conditions were also relevant: patients with coagulopathy (OR 2.29, 95% CI 1.17–4.44, p = 0.01), a history of neck surgery (OR 3.49, 95% CI 1.28–9.44, p = 0.01), or obesity (OR 2.10, 95% CI 1.24–3.56, p = 0.006) had a significantly greater likelihood of experiencing complications. Cervical immobilization was also associated with a markedly increased risk of serious complications (OR 4.68, 95% CI 1.15–19.09, p = 0.03). No significant associations were found with patient weight, respiratory index, platelet count, coagulation times (aPTT or INR), procedural technique, bronchoscopy, ultrasound, number of puncture attempts, or the professional performing the puncture. Capnography could not be reliably evaluated as an independent predictor due to complete separation, as no complications occurred among patients without capnography.

Additional laboratory and ventilatory parameters are provided in Tables S2–S4 (Supplementary Material). Of the 223 patients (43.2%) with technical difficulties, 88 developed complications (39%), of which 20 were classified as serious (8.9%). The original overlap of categories has been clarified.

### Other analyses

Complication rates showed a modest downward trend, from 32% in 2019 to 25% in 2022 (p = 0.08), suggesting a learning effect over time. Outcomes did not significantly differ between high-volume centers (performing >20 cases/year) and low-volume centers (<5 cases/year), although the latter contributed only 12% of total cases.

## Discussion

This multicenter study provides a contemporary overview of percutaneous bedside tracheostomy practices in Costa Rican intensive care units [14,15].

The median time to tracheostomy in this cohort was seven days of mechanical ventilation. However, the present study was not designed to evaluate early versus late tracheostomy as an exposure variable, and no stratified analysis by timing was performed [15,16,17,18]. Consequently, any discussion of early tracheostomy benefits reflects contextual evidence from prior studies rather than findings derived from this dataset.

Procedural practices were largely consistent with international standards. Previous studies have shown the procedure to be safe even in severe thrombocytopenia [19]; in our analysis, anticoagulation and coagulopathy were significantly associated with complications, but the majority of bleeding events were minor and controlled at the bedside.

Technical difficulties occurred in 43.2% of cases and were strongly associated with complications. In multivariable analysis, the presence of technical difficulty increased the odds of any complication more than fourfold, while serious technical difficulty was associated with a twofold increase in the risk of serious complications. The Griggs technique predominated, mainly due to equipment availability, although all staff were trained in both the Griggs and Ciaglia methods. Bleeding was the most frequent complication, mostly minor and managed with compression, while potentially life-threatening complications such as loss of airway, false tract, or bleeding requiring surgical intervention occurred rarely in this cohort. These rates are comparable to those reported in international series. Importantly, no significant differences were observed between specialists and supervised residents, supporting the role of structured training programs.

Airway management during the procedure was performed by physicians in 44.9% of cases and by respiratory therapists in 55.1%. Physician-led airway management was associated with higher odds of complications. However, this finding should be interpreted with caution, as operator assignment was not randomized and physicians were more likely to be involved in complex or clinically unstable cases, introducing potential confounding by indication. Therefore, this association is unlikely to reflect intrinsic differences in technical skill and instead underscores the importance of standardized training pathways and clearly defined competencies in airway management across professional groups.

Adjuvant tools were underutilized. Bronchoscopy was applied in only 3.1% of procedures, less than reported in systematic reviews, where bleeding rates varied depending on its use [8]. Ultrasound was used pre-procedurally in 17.7% of cases, but intra-procedural scanning was rare. International data suggest that ultrasound can identify vascular structures in up to 40% of patients and alter puncture site in up to 24%, supporting recommendations for its wider use [2, 20,21,22]. Capnography was employed in 74% of procedures. Although capnography was widely used, its independent association with complications could not be determined due to complete separation, as no adverse events occurred among patients without capnography, likely reflecting its preferential use in higher-risk patients. The variability observed among centers in our study echoes prior evidence that institutional practices and operator expertise are key determinants of outcomes [13]. Our observed rates of bleeding and airway-related events fall within the ranges reported in recent systematic reviews and expert updates [4].

No procedure-related deaths were observed in this cohort. This finding is consistent with reports from large series describing very low or absent procedure-related mortality, with published fatality rates of approximately 0.16%, most commonly associated with catastrophic bleeding or tracheal perforation [9,23].

Given the retrospective design, underreporting of minor adverse events is likely, potentially underestimating true complication rates. Furthermore, incomplete laboratory and ventilatory data limited adjustment for all potential confounders. Absence of long-term follow-up precludes evaluation of late complications such as tracheal stenosis. Our results align with recent multicenter analyses showing low major complication rates with percutaneous tracheostomy when performed by experienced teams. However, unlike some European cohorts, anticoagulation management practices differed, which may partly explain our observed bleeding rate. These differences underscore the need for cautious extrapolation.

Taken together, these findings support that percutaneous bedside tracheostomy can be performed safely in critically ill patients when conducted by trained ICU personnel. However, the risk of complications was higher in patients with coagulopathy, anticoagulation exposure, obesity, prior neck surgery, or cervical immobilization, highlighting the importance of careful patient selection and procedural planning. The broader integration of structured multidisciplinary training and adjunctive monitoring may further enhance safety in high-risk patients.

The results of this study are likely generalizable to similar middle-income ICU settings with established critical care training programs but may not directly apply to centers with limited access to adjunctive tools or where surgical tracheostomy remains the predominant approach. Additional multicenter data from Latin American ICUs are needed to further assess external validity.

This study has several limitations. Although data collection was prospective, the analytical approach was retrospective. No formal sample size calculation was performed, limiting statistical power for rare events. Heterogeneity across centers was substantial, with a large proportion of procedures concentrated in referral hospitals, and anticoagulation management was not standardized across ICUs, potentially confounding bleeding outcomes. Timing of tracheostomy was not analyzed as an exposure variable; therefore, the study cannot evaluate the impact of early versus late tracheostomy on procedural risk. Long-term follow-up was not available, precluding assessment of late complications. Finally, the near-universal use of capnography limited its evaluation as an independent predictor of complications, as complete separation precluded reliable estimation in multivariable models. Despite these limitations, this study represents the largest multicenter dataset on percutaneous tracheostomy in Central America and provides important epidemiological insights into current ICU practice.

## Conclusions

In this large multicenter cohort, most percutaneous tracheostomies were performed in neurosurgical ICUs of national hospitals, predominantly in male patients admitted for neurotrauma. Pneumonia, polytrauma, head trauma, intracranial hemorrhage, and postoperative neurosurgical care were the leading admission diagnoses, while prolonged or anticipated prolonged mechanical ventilation, neurological deficits, and failed extubation were the principal indications for tracheostomy, typically performed after a median of seven days of intubation.

Our findings showed that bleeding was the most frequent event, though the majority were minor and controlled conservatively. By applying a broad definition that included bleeding controlled by pressure, the overall adverse event rate reached 28.3%; however, serious complications occurred in only 3.9%, comparable to international series. Technical difficulties were common in patients with obesity, short neck, or coagulopathy, which were also associated with higher complication rates. Importantly, procedures performed by residents under supervision were not associated with increased risk.

These findings confirm that percutaneous bedside tracheostomy is a safe and effective procedure in critically ill patients when performed by trained ICU personnel. Risk stratification and the systematic use of adjuncts may support procedural safety, particularly in high-risk subgroups.

## Abbreviations

aPTT: Activated Partial Thromboplastin Time
BMI: Body Mass Index
CCSS: Caja Costarricense de Seguro Social
CI: Confidence Interval
CRF: Case Report Form
FiO_2_: Fraction of Inspired Oxygen
ICU: Intensive Care Unit
IQR: Interquartile Range
INR: International Normalized Ratio
OR: Odds Ratio
PPE: Personal Protective Equipment
SD: Standard Deviation
SOFA: Sequential Organ Failure Assessment
STROBE: Strengthening the Reporting of Observational Studies in Epidemiology

## References

[1] Fischler L, Erhart S, Kleger GR, Frutiger A (2000). Prevalence of tracheostomy in ICU patients. A nation-wide survey in Switzerland. Intensive Care Med [Internet].

[2] Raimondi N, Vial MR, Calleja J, Quintero A, Cortés A, Celis E (2017). Evidence-based guidelines for the use of tracheostomy in critically ill patients. J Crit Care [Internet].

[3] Durbin CGJ (2005). Techniques for performing tracheostomy. Respir Care.

[4] Rossi V, Binda F, Cordani C, Marelli F, Tammaro S, Colombo S (2025). Impact of tracheostomy on ICU stay in adult patients with ARDS: A systematic review. Intensive Crit Care Nurs [Internet].

[5] Ciaglia P, Firsching R, Syniec C (1985). Elective percutaneous dilatational tracheostomy. A new simple bedside procedure; preliminary report. Chest.

[6] François B, Clavel M, Desachy A, Puyraud S, Roustan J, Vignon P (2003). Complications of tracheostomy performed in the ICU: subthyroid tracheostomy vs surgical cricothyroidotomy. Chest.

[7] Worthley LI, Holt AW (1999). Percutaneous tracheostomy. Crit Care Resusc J Australas Acad Crit Care Med.

[8] Brass P, Hellmich M, Ladra A, Ladra J, Wrzosek A (2016). Percutaneous techniques versus surgical techniques for tracheostomy. Cochrane Database Syst Rev.

[9] Mahmood K, Wahidi MM (2016). The Changing Role for Tracheostomy in Patients Requiring Mechanical Ventilation. Clin Chest Med.

[10] Massick DD, Powell DM, Price PD, Chang SL, Squires G, Forrest LA (2000). Quantification of the learning curve for percutaneous dilatational tracheotomy. The Laryngoscope.

[11] Even-Tov E, Koifman I, Rozentsvaig V, Livshits L, Gilbey P (2017). Pre-procedural Ultrasonography for Tracheostomy in Critically Ill Patients: A Prospective Study. Isr Med Assoc J IMAJ.

[12] Ramírez Arce J, Padilla Cuadra J, Sánchez Arias M (2009). Traqueostomía percutánea por dilatación: Reporte de 70 casos. Acta Médica Costarric [Internet].

[13] Merola R, Iacovazzo C, Troise S, Marra A, Formichella A, Servillo G (2024). Timing of Tracheostomy in ICU Patients: A Systematic Review and Meta-Analysis of Randomized Controlled Trials. Life [Internet].

[14] Argüello Quirós MF, Salas-Segura D (2016). Mortalidad de pacientes de una unidad de cuidados intensivos. Un estudio prospectivo de 12 meses. Rev Médica Univ Costa Rica.

[15] Hosokawa K, Nishimura M, Egi M, Vincent JL (2015). Timing of tracheotomy in ICU patients: a systematic review of randomized controlled trials. Crit Care [Internet].

[16] Elkbuli A, Narvel RI, Spano PJ, Polcz V, Casin A, Hai S (2019). Early versus Late Tracheostomy: Is There an Outcome Difference?. Am Surg.

[17] Bösel J (2017). Use and Timing of Tracheostomy After Severe Stroke. Stroke.

[18] McCann MR, Hatton KW, Vsevolozhskaya OA, Fraser JF (2020). Earlier tracheostomy and percutaneous endoscopic gastrostomy in patients with hemorrhagic stroke: associated factors and effects on hospitalization. J Neurosurg.

[19] Kluge S, Meyer A, Kühnelt P, Baumann HJ, Kreymann G (2004). Percutaneous tracheostomy is safe in patients with severe thrombocytopenia. Chest.

[20] Rees J, Haroon Y, Hogan C, Saha S, Derekshani S (2018). The ultrasound neck imaging for tracheostomy study: A study prompting ultrasound screening prior to percutaneous tracheostomy procedures to improve patient outcomes. J Intensive Care Soc.

[21] Simon M, Metschke M, Braune SA, Püschel K, Kluge S (2013). Death after percutaneous dilatational tracheostomy: a systematic review and analysis of risk factors. Crit Care Lond Engl.

[22] Osman A, Sum KM (2016). Role of upper airway ultrasound in airway management. J Intensive Care.

[23] Chung W, Kim BM, Park SI (2016). Simply modified percutaneous tracheostomy using the Cook^®^ Ciaglia Blue Rhino^TM^: a case series. Korean J Anesthesiol.

